# First responders’ experiences with major earthquakes in Türkiye: a qualitative study of innovation needs and challenges

**DOI:** 10.1186/s12873-025-01217-9

**Published:** 2025-04-14

**Authors:** Zeynep Sofuoglu, Aysel Başer, Turhan Sofuoglu, Ömer Faruk Sönmez

**Affiliations:** 1 Medical Faculty, Department of Public Health, Izmir Demokrasi University, Izmir, Türkiye; 2 Medical Faculty, Department of Medical Education, Izmir Demokrasi University, Izmir, Türkiye; 3 Emergency, Disaster, Ambulance Physicians’ Association, Izmir, Türkiye; 4 School of Medicine and Population Health, University of Sheffield, Sheffield, UK

**Keywords:** Disaster management, First responders, Innovative technologies, Teamwork, Communication, Collaboration

## Abstract

**Background:**

The response phase is a phase of disaster management that begins when a disaster occurs. The experience of the First Responders who responded in the first days to the 2023 earthquake in Turkey, which killed more than fifty thousand people, is expected to inform and add value to the preparations for subsequent disasters.

**Methods:**

A qualitative approach was used; focus group interviews were conducted with 15 first responders (8 fire fighters and 7 medical personnel) that responded in the first moments of the devastating Kahramanmaraş and Hatay earthquakes. The interviews conducted in June 2023. A qualitative approach with the thematic analysis method was employed.

**Results:**

Based on the analyses the research six main themes and 13 subthemes were identified. The identified themes were resources, needs, collaboration, innovation, disaster management and challenges. The participants emphasized the need for better communication tools, protective equipment, and technologies such as drones and advanced sensors to enhance disaster response efforts. The findings also highlight the critical role of collaboration between different organizations and the necessity for integrated disaster management practices and applications. The findings obtained from the views of experienced first responders will inspire national and international disaster crisis managers, policy makers and technology providers to develop more effective disaster response methods. Through technological solutions and better training, the safety and effectiveness of first responders can be significantly improved in future disaster scenarios.

**Conclusions:**

This study fills an important gap in the literature by investigating the innovation needs and challenges faced by first responders during the 2023 earthquakes in Türkiye. In this study, the gap analysis was determined through interviews conducted with teams that responded to the 2023 major Turkey earthquake in the initial hours following the disaster. These findings are of significant value in guiding the improvement of the approaches and development of technological products in the field of disaster management.

**Supplementary Information:**

The online version contains supplementary material available at 10.1186/s12873-025-01217-9.

## Introduction

The immediate actions of first responders are crucial for saving lives, but these efforts often face significant operational challenges due to resource limitations, communication barriers, and insufficient technological integration. Research highlights that effective disaster response requires real-time situational awareness, interoperability across organizations, and robust safety measures for responders [1].

On February 6, 2023, Türkiye was struck by two powerful earthquakes, measuring 7.7Mw and 7.6Mw, centred in Kahramanmaraş, followed by a 6.4Mw tremor in Hatay on February 24 [2]. This seismic sequence, one of the region’s most severe in the last century, caused widespread destruction, including surface deformations such as landslides, liquefaction, and cracks [3, 4].

The impact of these earthquakes extended across eleven provinces, affecting approximately 14.013 million people—16.4% of Türkiye’s total population [5]. This disaster covered an area of 108,812 square kilometres, surpassing the size of some countries, and resulted in over 50,500 deaths, 100,000 serious injuries, the destruction of half a million structures, and extensive damage to critical infrastructure such as telecommunications, transportation, healthcare, water, and energy systems [2, 6].

First responders face various occupational hazards including vehicle crashes, physical injuries, and the extreme demands placed on their musculoskeletal systems, as well as exposure to hazardous materials, stress, erratic work hours, and extreme temperatures [7]. While the nature of these incidents is well-documented, the response and associated risks can vary significantly, making the identification of risks and the characterization of hazards essential [8]. Moreover, the regular participation in disaster response efforts can lead to casualties among first responders themselves. Consequently, disasters exert considerable pressure on the mental health of both civilians and first responders [9].

Developers and technology providers must be attuned to the innovation needs and gaps identified by first responders, with proposed solutions aimed at closing these gaps and enhancing their safety and efficiency [10].

In an era where user-centered innovation is increasingly prevalent, fuelled by advances in computing and communication technologies, Previous frameworks, such as those proposed by the International Forum for Advanced First Responder Innovation (IFAFRI), have identified critical areas for improvement in disaster response. Key gaps include the need for real-time tracking of responders, immediate detection of threats and hazards, and the ability to manage and integrate data from multiple sources during a crisis [11].

The present study aims to address a significant gap in the extant literature by exploring the innovation needs and challenges experienced by first responders during the 2023 earthquakes. The central research question guiding this study is as follows: What are the innovation needs and operational gaps faced by first responders during large-scale disasters? By focusing on the frontline experiences of responders in Kahramanmaraş and Hatay—regions that were severely affected by the earthquakes—this research provides practical insights for improving disaster preparedness and response.

It is a well-documented issue within the relevant literature that populations affected by health emergencies often experience elevated levels of illness, disability and mortality, often due to inadequate access to timely, effective and quality health services [12]. The present study has been designed to address this gap. Gap analysis studies in the literature are generally obtained by examining past events or possible scenarios within the scope of group studies conducted with experienced first responders. However, this study is of significant value as it reveals the findings of gap analysis based on interviews with the First Responders working in the field during the first moments of the Major 2023 Türkiye earthquakes.

The objective of this research is to ascertain the challenges and innovation requirements of the First Responder teams that responded to the 2023 Kahramanmaraş and Hatay Earthquakes at the initial phase. By identifying and addressing these needs, this research contributes both to academic discussions and operational improvements in disaster management practices.

## Materials and methods

This study was designed as a thematic analysis to explore the challenges, gaps, and innovation needs faced by first responders in the aftermath of the earthquakes in Türkiye, with the goal of proposing technical solutions to address these gaps. A qualitative research design was employed, utilizing focus group interviews as the sole data collection method to capture diverse perspectives and experiences [13, 14]. Although qualitative content analysis is commonly used for analysing textual data, this study employed thematic analysis to allow for a more flexible and interpretative approach to understanding first responders’ experiences. Unlike qualitative content analysis, which emphasizes the systematic categorization of manifest content, thematic analysis enables the identification of underlying themes and patterns, offering a richer exploration of emergent concepts within the data [15, 16]. Thematic analysis was chosen due to its suitability for structured interview forms, which provide predefined topics while still allowing for inductive theme development. According to Braun and Clarke [15], thematic analysis is particularly effective in studies that aim to identify both predefined structure and emergent insights, making it an appropriate methodological approach for this research [15].

### Participant selection and data collection

Participants for this study were selected through purposive sampling which targeted first responders who actively participated in the earthquake response efforts in Kahramanmaraş and Hatay provinces. The selection process involved close coordination with local emergency management agencies and organizations to identify individuals with direct involvement in rescue operations. Potential participants were approached through formal invitations sent via their respective organizations, ensuring that they were fully informed about the study’s purpose and requirements. To mitigate potential biases due to sole reliance on willing participants, efforts were made to ensure diversity in the sample by considering variables such as professional role (e.g., paramedics, firefighters, search and rescue specialists), experience level, and geographic assignment. Additionally, participants were briefed on the importance of candid responses to reduce self-censorship and promote authentic feedback. All participants provided informed consent, ensuring ethical compliance and voluntary participation [17].

Focus group interviews were conducted as the primary data collection method. This approach is particularly valuable in qualitative research for exploring complex behaviours, attitudes, and experiences through group dynamics and interactions [18]. Participants were organised into three focus groups, each comprising five individuals, to ensure depth and richness in the data collected while allowing for manageable group discussions. Following principles are considered ideal suggested by Morgan & Spanish (1984) [19], Onwuegbuzie et al. (2009) [20], O. Nyumba et al. (2018) [21]:

- For each research, at least 3 different groups must be constructed.
- Each group should consist of 4 to 12 people.
- Interviews should take at least 1–2 h.
- Quality of data must be saturated.

The interviews were conducted face-to-face to ensure direct interaction, foster engagement, and capture non-verbal cues more effectively, as this method was deemed essential for understanding the firsthand experiences of first responders. Each session was facilitated by experienced first responders, with researchers in attendance to observe, take notes, and ensure the smooth progression of discussions. The interviews took place between June 12th and 22nd 2023, lasting approximately 75 min each.

### Structured interview guide

The structured interview guide covered key topics, including first responders’ challenges, communication issues, technology use, situational awareness, teamwork, protective equipment, and innovation needs in disaster response.The Supplementary Material 1 presents the Focus Group Discussion Form.

### Data analysis

The focus group discussions were audio-recorded with participant consent and transcribed verbatim. Thematic analysis was applied to identify patterns and themes relevant to the study objectives using Braun and Clarke’s [15] six-step framework [22]. The analysis followed an iterative process, involving an initial reading of transcripts, open coding, and theme generation, and refinement to ensure emerging themes were refined and validated through continuous comparison [14, 15, 23]. Two independent researchers conducted the coding process, and discrepancies were resolved through structured discussions to enhance reliability.

### Achieving data saturation

The study aimed to reach data saturation, defined as the point where no new codes or themes emerged from the data [24, 25]. This study employed Graneheim and Lundman’s (2004) qualitative content analysis approach, ensuring a structured and systematic analysis of the data. An iterative process of data collection and analysis was followed, where each focus group discussion was transcribed verbatim and systematically coded. Meaningful units were identified, condensed, and categorized into themes. Two independent researchers conducted the coding process to enhance reliability, with discrepancies resolved through structured discussions [24, 26]. Thematic refinement was carried out through continuous comparison and inductive analysis, ensuring that emerging themes accurately captured first responders’ experiences and innovation needs. Additionally, researcher debriefings and cross-checking of coded data were conducted to further validate the findings and ensure methodological rigor [23–25].

### Addressing bias in facilitation

Given the use of experienced first responders as facilitators, the research team implemented measures to mitigate potential biases. Facilitators were trained to remain neutral and avoid leading questions. The presence of a secondary observer during discussions ensured consistency and monitored for any undue influence on participant responses [27].

### Ensuring trustworthiness in qualitative research

To ensure the trustworthiness of this qualitative study, we followed Lincoln and Guba’s (1985) criteria, incorporating credibility, transferability, dependability, and confirmability into our research process [26]. Additionally, thematic analysis was supported by detailed documentation of the coding process, including codebooks and excerpts from the data to illustrate key themes.

Credibility: Member Checking; Participants were provided with transcripts to verify the accuracy of the data. Peer Debriefing: An independent expert reviewed the research process and findings. Consistency Checks: While inter-coder reliability metrics were not numerically reported, consistency checks were conducted through research team meetings and discussions to strengthen interpretative validity.

Transferability; Thick Description; Detailed contextual descriptions were documented, allowing for comparison across different settings. Collaborative Validation, the research team conducted regular meetings, clarified research boundaries, and secured ethical approval. All documents were stored on a shared cloud platform for accessibility.

Dependability; External Peer Review; An experienced physician from the Emergency Disaster and Ambulance Physicians Association reviewed the findings and provided revision suggestions.

Confirmability; Transparent Documentation; All records and decisions were systematically archived in a shared cloud storage system accessible to all researchers. Secure Data Storage: Ethical considerations included maintaining participant confidentiality through anonymized data and secure storage of recordings and transcripts.

These measures enhance the rigor, reliability, and transparency of our study, ensuring that findings accurately reflect participants’ experiences and are methodologically sound.

## Findings

The analysis of qualitative data derived from focus group interviews with first responders who participated in the earthquake rescue operations in Kahramanmaraş rovides valuable insights into their experiences, challenges, and the innovation needs that emerged in the aftermath. The demographic profile of the participants shows a clear predominance of male respondents (86.67%, *n* = 13), with an average age of 42 years (range: 31–65). The professional background of participants was predominantly in firefighting (53.3%, *n* = 8), with the remainder being medical providers. Engagement in the earthquake rescue efforts was distributed across the initial days following the disaster, highlighting a sustained response effort (Table 1). All participants reported previous experience earthquakes in their lives.

**Table 1:** Participant demographics

Participant code	First responder type	Gender	Years worked	Number of working days in the earthquake zone	Participants’ earthquake related events history
P1	Fire fighter	Male	10	10 days	Present
P2	Fire fighter	Male	9	9 days	Present
P3	Fire fighter	Male	10	13 days	Present
P4	Fire fighter	Male	10	13 days	Present
P5	Fire fighter	Male	10	13 days	Present
P6	Fire fighter	Male	10	13 days	Present
P7	Physician	Male	35	3 days	Present
P8	Physician	Female	25	6 days	Present
P9	Paramedic	Female	15	6 days	Present
P10	Paramedic	Male	15	6. days	Present
P11	Paramedic	Male	10	9 days	Present
P12	Fire fighter	Male	15	9 days	Present
P13	Paramedic	Male	35	7 days	Present
P14	Fire fighter	Male	15	10 days	Present
P15	Physician	Male	15	6 days	Present

This section synthesises the findings from the thematic analysis of the interview transcripts, with the aim of revealing the specific needs and challenges faced by first responders during the earthquake response. The analysis yielded six primary themes, accompanied by 13 sub-categories and 59 codes which summarise the core aspects of the responders’ experiences and the critical areas for innovation and improvement (Table 2).

**Table 2:** An overview of themes, subthemes and codes extracted from the data analysis

Themes	Subthemes	Codes
Theme 1: Resources	Communication network (infra structural level)	- Limited radio communication - WhatsApp communication - Drone signal loss due to jammer usage - Lack of communication between teams at disaster sites
	Protective equipment	- Firefighter suits, masks, gloves, and googles - Firefighter gear preference - Boot soles melting due to extreme conditions - Goggles becoming unusable due to dust - Need for stronger helmets - Need for disposable suits
Theme 2: Needs	Search and rescue	- Damage assessment using drones - Identifying survivors using thermal cameras - Structural collapse risk - Noise interference in detection - Lack of devices to precisely locate people under rubble
	Emergency response	- Shortage of field hospitals - Inadequate emergency facilities - No system for patient registration - Insufficient number of ambulances - Shortage of medical supplies and equipment
Theme 3: Collaboration	Communication	- WhatsApp-based coordination - Multi-agency coordination - Unclear authority distribution in the field - Mismatch in rescue techniques among different teams - Lack of a structured task assignment system - Dividing into separate teams to improve efficiency - Delays due to leadership changes before the disaster
	Information sharing	- AFAD-UMKE (teams) contact information sharing - Lack of information sharing within the FR teams - Absence of records on building occupants - Need for a centralized communication hub - Need for interoperability
Theme 4: Innovation	Technological advancements	- False positive thermal detection - No GPS tracking devices for FR rescuers - Need for durable drone lenses - Need for wearable safety gear
	Situational awareness	- Absence of gas leak detection devices - Lack of night vision in drones - Devices needed to differentiate between living and deceased victims
Theme 5: Disaster management	Logistics	- Need for a coordination hub - Need for regional logistics hub - Road blockages hindering rescue and transport - Transportation difficulties
	Coordination	- Lack of task allocation - Inefficiency in managing teams arriving from different cities - Lack of pre-disaster building data
Theme 6: Challenges	Occupational health and safety	- Sharp metal debris causing injuries - Gas leaks in work areas - Dust and debris leading to eye infections - Encountering death threats
	Psychological and Emotional Stress	- Emotional impact of children casualties and death bodies - Feeling guilty about not being able to save earthquake victims - Witnessing sadness of people searching for relatives - Psychological pressure from continuous exposure to death and casualties - Heavy emotional burden on rescue teams
	Physical needs	- Cold weather and rain affecting rescue operations - Shortages of food and water - Exhaustion from long working hours - Inadequate protective and durable clothing

The following themes provide a comprehensive overview of the areas where innovative solutions could significantly impact the efficacy and safety of first responders in disaster scenarios. The Supplementary Material 2 provides sample participants statements related to all identified themes and codes.

### Theme 1: Resources

The first theme to emerge from the analysis concerns the resources used during the earthquake response. Participants highlighted the critical role of various types of resources, including drones, thermal cameras, audio recorders and communications equipment, in the effectiveness of operations. The need for improved communications networks and technology was highlighted as a means of improving operational efficiency. Issues of loss of connectivity and network weaknesses were identified as contributing to communication and coordination challenges, primarily due to latency in location information. The need to establish a redundant network during disasters was emphasised to mitigate these issues.

*P2… Sometimes the military would activate jammers for security reasons. This prevented us from communicating with the drones. We should be able to continue with other methods in such cases*.

P10 *We only had mobile phones. The messages came through WhatsApp. However, due to network problems, the location information either did not come or came late.*

Furthermore, the use of protective equipment—such as helmets, firefighter outfits, glasses, masks, and gloves—was noted as essential for enhancing the safety of first responders. However, a reported shortage of resources indicated that not all participants had adequate access to necessary equipment. The enhancement and ruggedization of equipment, particularly those identified by participants, were recommended to ensure their effectiveness and adaptability to disaster contexts. Additionally, the importance of distinct and recognizable uniforms for rescue teams was mentioned to avoid confusion and facilitate easy identification of first responders in the field.

P4 *We used helmets, fire suits, goggles, masks and gloves. The suit must be in two pieces for toilet use. Helmets must be undamaged. It is not safe to reuse damaged ones. Boot soles can melt. Goggles are useless because of the dust.*

### Theme 2: Needs

The analysis identified several needs expressed by first responders in emergencies that go beyond the common requirements of rescue and medical teams, such as communication, coordination, the importance of having decision support technologies such as artificial intelligence, augmented reality solutions that show the undamaged building before the earthquake struck in order to make a better decision about entering the damaged structure to rescue people trapped in the rubble.

P2 *The building was in danger of collapsing. It was not safe, and no one could analyse it. So, we could not enter it. If we could determine the right place to drill a hole and get inside, we could rescue the people. The building was unrecognisable even to the people in the neighbourhood.*

Medical first responders emphasised the need for increased hospital capacity, the establishment of field hospitals, access to medical equipment, logistics management and electronic record systems. In contrast, rescue teams highlighted the need for improved technological support through cameras, drones, radios and other devices to assist rescue operations. A critical concern for all first responder groups was the lack of personnel and security measures available to meet the demands of large-scale disaster response.

P8 *In such huge disasters, medical care cannot be accomplished without 100 bed capacity field hospitals with EMT3 level training.*

P9 *We immediately started treating the injured, but there was no record of when and how the patients arrived.*

### Theme 3: Collaboration

The analysis revealed collaboration as a crucial theme, with a particular emphasis on the collaborative efforts observed among national teams in the field. Participants underscored the significance of inter-organizational collaboration, highlighting how diverse entities such as UMKE (National Medical Rescue Team), AFAD (Disaster and Emergency Management Authority), the Disaster Coordination Center (AKOM), municipality personnel, volunteer groups, and non-profit organizations pooled their skills and resources for effective disaster response.

P7 *In the plane, there were AFAD, UMKE, Turkish Radio Amateur Society (TRAC) and military search and rescue teams. We landed on İncirlik Airbase at 16:30 next day.*

Effective collaboration was facilitated by robust communication and information sharing practices. Tools such as mobile phones, instant messaging applications and inter-organisational communication channels played a key role in these collaborative efforts. The exchange of contact information, timely updates from AFAD, and the use of instant messaging for coordination were all cited as instrumental in improving operational efficiency. The presence of multiple communication channels was seen as essential to meet the dynamic challenges of disaster response.

P11 *I am a UMKE staff member. In the Adıyaman centre we were included in the UMKE Medical Endpoint. There was AFAD, international and national volunteer teams who gave us an address and we went to the rubble zone. We worked in two teams of 5 people each.*

However, challenges were identified in aligning the protocols and procedures of different organisations. For example, some participants reported occasional conflicts between civilian rescue teams and public first responder organisations, highlighting areas where further coordination and understanding could improve collective response efforts.

P3 *… we knew the team we were working with, we knew our skills and techniques, but when a civilian rescue team arrived, sometimes we could not cope with their techniques….*

### Theme 4: Innovation

Participants proposed innovative solutions to improve response capabilities and address challenges. Key suggestions included the establishment of mobile or online coordination centres, the use of building security monitoring systems and the use of advanced dispatching services. The active use of drones, along with robust solutions, sensors, wearable technology and external building scanning tools were recommended for more effective rescue efforts.

Innovations aimed at distinguishing living from deceased victims, facilitating triage and accurately monitoring vital signs were highlighted. Among these, remote measurement of vital signs was noted as a potential method to reduce misleading factors associated with thermal imaging.

P2 *Using a drone equipped with a thermal camera, it took 4–5 hours to rescue a human-shaped object emitting heat. Then it was understood that the object was not a baby, but a teddy bear. The situation caused a loss of time. It was understood that detecting the heat wave and shape was not enough. It looked like the silhouette of a baby, but it was a fibre heated by the sun. It was not enough to just detect the shape and heat. There is a need to add sensors that can detect parameters such as breathing rate, heartbeat or body movement.*

P8 *The innovative triage systems must take photos, make identification, measure vital signs, and show patients’ location. I think medical first responders shall have nasal end-tidal CO2 measurement devices. It shows everything. Massimo has such a device. The bigger ones also perform hemogram analysis. Hundreds of patients can be saved with these devices. Identification is very important; hospitals should have put barcoded bracelets to the saved victims.*

P2 *… The damage assessment was done with drones within 15 minutes, whereas the area was discovered after 5–6 hours of pedestrian assessment. First, we did an aerial survey of the area. At the first stop, we could not see any living people, but we could see the dead. Drones were very useful to see the big picture.*

Health and safety innovations were also highlighted, such as durable protective equipment, wearable technology with GPS tracking, and chemical gas detection systems to identify hazards and ensure the safety of first responders while working in the rubble.

P11 *When we entered the ruins, the safety of the building could not be guaranteed. We could not feel safe during the aftershocks. If the drones had been used effectively, the damage to the area could have been assessed and a faster rescue operation could have been carried out by using thermal cameras to locate people under the rubble.*

P 12 *The GPS trackers are necessary because when you send the team into the zone, there is always a risk of them being trapped under rubble.*

### Theme 5: Disaster management

Participants’ assessments of disaster management practices were predominantly negative. Key issues identified included a significant lack of coordination, effective communication and reliable information in the first hours after the disaster.

The inefficiency of communication and information sharing between different organisations was highlighted as a major obstacle to effective disaster response coordination. It was noted that critical information often came from residents or was missing altogether, rather than being systematically disseminated by coordinating agencies. This lack of timely and reliable information hampered the coordination of resources, management and execution of search and rescue operations.

P7 *I think there should have been a coordination headquarter from centre to the farthest edge, especially in Adana. Even if it was online or mobile….*

P8 *…. The ‘core management team’ needs to know all the resources, roads, airports of that province and this needs to be prepared in advance. These will support the deployed teams to manage disasters*.

P9 *…. Cold, rain, crowd, lack of coordination, large number of injured, lack of tools and medical equipment, lack of electronic registration system, lack of collaboration with professional rescue teams….*

In addition, the specific communication and information requirements of first responders varied significantly between organisations. For example, search and rescue teams emphasised the need for 3D models of buildings and information on the occupancy of these structures, while medical first responders required access to patient records, addresses, hospital emergency plans, capacity and verification of the identity of volunteer medical personnel. This diversity of needs underscores the complexity of disaster management and the critical importance of tailored, rapid information sharing and coordination mechanisms.

P12 *We should have seen the 3D models of the original buildings at that moment. The sounds were mixed up, we could not tell which building was which and where we were in the original building… Search and rescue teams need to have access to a database of this information. We did not know how many people were in the building, how old they were, what their names were, or any information about the building. It was such a mess.*

### Theme 6: Challenges

The “Challenges” theme summarises the wide range of occupational health and safety issues faced by first responders. The main concerns were categorised under hazards, safety and occupational accidents. Participants described a variety of hazards:

- Physical: including aftershocks, earthquake effects, collapsing rubble, noise, dust, extreme cold and adverse weather conditions.
- Chemical: Exposure to toxic and flammable substances.
- Biological: Risks from rotting food, corpses, micro-organisms and infectious diseases.
- Ergonomic: Risks from difficult working environments, poor lighting and unsuitable clothing.
- Psychosocial: Covers long hours, stress and the emotional toll of rescuing or recovering victims, especially children.

P2 *In the areas where we were working, there could be a gas leak that could lead to an explosion. You might not be able to smell it because you can’t smell anything because of the corpses and the intense carbon dioxide. Not only corpses, but also rotten food, exploded fridges and meat start to smell… Cold, rain, construction machinery, heat-emitting pieces of glass made things more difficult.*

A notable aspect of the psychosocial challenges was the verbal aggression and threats from relatives of victims, which created additional stress and danger for responders. Such situations often escalated when relatives used dangerous methods to draw attention to their loved ones believed to be trapped under the rubble. In addition, there were reports of injuries to first responders caused using drilling or cutting tools during rescue operations.

P12 *Damage caused by iron bars, injuries caused by tiles, cuts caused by glass were too frequent… Long working hours, infections of the eyes caused by dust and particles, impaired eyesight, blocked ears, especially enervation caused by children’s bodies, stress…*.

Another major concern was the need for improved security measures. Responders called for increased physical protection and law enforcement presence to protect both responders and affected communities in the chaotic aftermath of the disaster.

P1 And there was a relative of a tribal member who said, ‘If you bring any construction equipment, we will k*** you’. This risk also arises because they think their relative is alive under the rubble.

Within the theme of “Challenges”, emotional distress emerged as a significant sub-category, underlined by participants’ recounting of harrowing experiences marked by profound grief. These included the distressing sight of injured or deceased children, the desperate cries for help from those trapped under the rubble, and the confusion and helplessness caused by multiple, simultaneous voices emanating from the rubble. First responders reported increased stress levels due to the anticipation of the arrival of ambulances for the injured and the constant calls for help from victims and their anxious families. Confrontations and threats from victims’ relatives were highlighted as particularly stressful encounters, adding to the already significant emotional burden of first responders.

P3 *We were trying to drill a gallery and get into the rubble and a man came and said: ‘We have an injured relative over there, you are working here but there are people out there. People came and said, ‘I have a child over there’, the other one came and said, ‘My spouse is over there’, if we look at both situations, please decide which one we should go to….*

Participants also emphasised the critical importance of Maslow’s hierarchy of needs - such as air, water, food and sleep - in maintaining the well-being and operational effectiveness of first responders. The long working hours reported by most participants posed a significant challenge to maintaining physical and mental energy for prolonged rescue operations. This aspect highlights the fundamental need to address basic physiological and safety needs to ensure that first responders can perform their duties effectively and sustain their efforts during prolonged disaster response operations.

## Discussion

The 2023 earthquakes in Kahramanmaraş and Hatay have resulted in over 50,000 deaths and more than 100,000 casualties, highlighting the urgent need for innovative solutions to support first responders.

### Theme 1: Resources

The utilization of innovative technologies and communication tools can enhance the efficiency of first responder operations. Technologies such as drones, thermal cameras, and sound recorders play a crucial role in locating survivors and assessing damage [28]. A robust communication infrastructure, coupled with reliable internet connectivity, facilitates effective coordination and rapid data exchange among various units. The combination of innovative technologies, protective gear, and strategic coordination significantly improves the safety and effectiveness of rescue operations. Protective equipment is essential for ensuring the health and safety of first responders. Choosing the right equipment helps protect against environmental risks [29]. Moreover, the colour compatibility and visibility of uniforms and protective gear enable quick identification among different groups of first responders [30].

### Theme 2: Needs

This study highlights the critical needs and challenges faced during disasters. Obstacles such as inadequate communication and coordination, security lapses, strained emergency services due to impacted hospitals, transportation issues (to the incident field of the FRs and from to field to the hospitals of the casualties), and the lack of electronic registration systems significantly hinder effective search and rescue operations [31]. Measures must be implemented to address these challenges and ensure swift transport to disaster zones. In the chaotic environment typical of disasters, medical first responders require efficient tracking systems to monitor and administer effective treatment. Paper based triage tags are not easy to apply. To meet these demands, the deployment of advanced and innovative technologies is essential. Reducing barriers to effective patient information sharing is a critical need during disasters. The novel solutions that ease this responsibility and leverage existing infrastructure should be explored [32].

Medical surge preparedness should be an integral and essential part of hospital preparedness as well as health system programs, especially in disaster-prone countries, to cope with disasters and emergencies by reducing the losses of lives and disabilities [33].

World Health Organization recommended to establish a surge system at country level to facilitate rapid mobilization and requests for assistance if/when neede; in the EMT 2030 Strategy [12].

### Theme 3: Collaboration

In Türkiye, the collaboration among governmental agencies, non-governmental organizations, local authorities, and the industry sector plays a significant role in disaster response and resilience. First responders must collaborate closely with all relevant organizations within the disaster zone. Collaboration protocols, training, and drills are crucial for fostering national and international teamwork, which is essential to avoid conflicts arising from differing procedures among various organizations. Effective communication forms the foundation of this collaboration. During earthquake response efforts, collaboration among first responders has been facilitated by mobile phones, instant messaging applications, and inter-organizational communication networks. The SENDAI Framework Programme also advocates for collaboration between national and international organizations and emphasizes the importance of drills to enhance disaster resilience [34].

### Theme 4: Innovation

The importance of innovation has been underscored by recent earthquakes, which have exposed gaps in coordination and information flow among local authorities especially with the use of social media and spread of disinformation [35]. For example, the safety of rescue teams entering compromised structures can be ensured through rapid and detailed analysis of building integrity. Although drones equipped with thermal cameras can significantly enhance the efficiency of rescue efforts, it is important to recognize that thermal imagery can be misleading, necessitating consideration of additional factors during search operations [36]. Advances in sensor technology and integration with data analytics can lead to more effective operations. Innovation is also critical for hospitals to withstand the chaos of disasters. The development of mobile or online coordination centres allows hospitals to share resources and capabilities, enhancing inter-hospital collaboration. Additionally, training hospital staff to use wearable devices can expedite triage processes and improve patient management [37].

### Theme 5: Disaster management

Occupational health and safety responsibilities during disasters primarily fall to governmental organizations, followed by local authorities. As these entities fulfil their roles in maintaining occupational health and safety, each first responder must also take personal responsibility for their own safety. Organizations are tasked with training first responders, providing equipment, assessing risks, and implementing protective measures. Local authorities are responsible for conducting these assessments and implementing measures on the ground. Individual responsibilities include participating in training, properly using protective equipment, adhering to protocols, and reporting hazardous conditions. Failure to prepare for disaster may be due to a lack of knowledge and skills in that population despite high intentions [38]. In the context of a disaster affecting 11 cities with a total population of 14 million, it has been observed that there was a lack of sufficient equipment and local leaders were unable to adequately assess situations and risks, leading to inappropriate response measures [39].

### Theme 6: Challenges

The protection of first responders’ health is paramount in disaster operations. While advanced protective equipment can reduce exposure to hazards, additional measures are necessary to support the physical and mental well-being of first responders. Abuse against the emergency medical services was formed from two categories, namely misuse of emergency medical services by individuals and interference of the people and patient’s family in relief process [40]. Our findings showed that FR faced emotional stress after responding to the 2023 Kahramanmaraş Earthquake, Khazaei et al., who used qualitative approach found out majority of EMS personnel stressed due to the unknown and unpredictable conditions of the scene and Afshari et al. showed that FRs worry about the potential threats to their life [41, 42].

Innovative solutions are also needed to enhance equipment and tools with advanced technology. For example, lightweight portable devices are more manageable in disaster zones. Robotic solutions could potentially replace humans in hazardous areas that require exploration [43]. The amount of personal protective equipment was a significant concern noted during interviews, along with the challenges posed by the smell of dead bodies, dust, and their effects on operations. Enhancements to equipment should focus on ergonomic design, lightweight materials, ruggedization, and integration of smart technologies. Such improvements, along with the use of wearable and drone-mounted sensors, can make operations more efficient, secure, and comfortable. Additionally, the ability to detect structural risks and locate survivors using drone-borne technologies can greatly increase the overall efficiency of operations while reducing casualties.

As the integration of advanced technologies in enhancing disaster response capabilities is analysed, it is crucial to highlight specific projects and good projects that have been recognized as best practices within the European framework.

The European Commission has consistently underscored its commitment to bolstering disaster-resilient societies through its Civil Security for Society Work Plan. Notably, during the 2018–2020 period, there was a distinct call for advancements in technologies aimed at augmenting the security and efficiency of first responders, as well as enhancing victim detection technologies. Reflecting this priority, the Commission has allocated substantial funding, supporting 14 projects with a combined budget of 90 million euros. Prominent among these initiatives are the TeamAware project, which integrates Artificial Intelligence and Augmented Reality to boost team situational awareness, and the ASSISTANCE project, which develops adapted situation awareness tools and tailored training scenarios to increase capabilities and enhance the protection of first responders [44].

The focus of the TeamAware and ASSISTANCE projects is to elevate the situational awareness and security of first responder teams by providing sophisticated decision support systems. Moreover, the requirements and feedback from first responders within the consortium have been pivotal in shaping these projects. The TeamAware project, for instance, has significantly contributed to enhancing in-team, environmental, and social situational awareness [45]. The deployment of wearable devices and advanced sensory technologies, such as drone-borne chemical and acoustic sensors, plays a critical role in identifying and mitigating potential threats, thereby securing both first responders and civilians effectively. Further, the Team Monitoring and Infrastructure Monitoring Systems developed under TeamAware have been instrumental in enhancing the coordination and safety of first responders during operations, particularly in earthquake scenarios prone to aftershocks.

The IProcure Security PCP project represents a significant endeavour under the auspices of the European Commission’s Civil Security for Society Work Plan [46]. This project aims to revolutionize the triage process during disaster scenarios through the development of an Innovative Triage Management System. Funded amidst a focus on enhancing technologies for first responders during the 2018–2020 period, the IProcure project received a portion of the total 90 million euros allocated to support 14 projects aimed at increasing responder security and improving victim detection technologies.

The Innovative Triage Management System introduced by IProcure is designed to expedite and refine the assessment and care of injured individuals. It incorporates decision support tools that optimize resource management and improve communication and coordination with Emergency Medical Services [46]. By reducing ambulance transport times and offering targeted training, the system enhances the overall efficiency of emergency medical responses [47]. Furthermore, it includes features that keep families informed post-evacuation, thus adding a layer of psychological comfort during crises, which is crucial for comprehensive disaster management [48].

These projects not only address critical gaps in emergency medical services but also exemplifies the proactive approach encouraged by the European Commission to integrate advanced technological solutions into first responder operations. The focus on innovative and practical applications ensures that emergency responders are equipped with the necessary tools to manage and mitigate the challenges presented by major disasters effectively.

During the 2023 earthquakes, responders encountered many of these challenges firsthand. For instance, the difficulty in maintaining clear communication across different organizations and the lack of sufficient protective equipment limited their ability to operate effectively. Additionally, the absence of integrated systems for real-time information gathering and sharing further complicated rescue operations [49].

### Recommendations

In response to the challenges identified through focus group interviews and aligned with the thematic insights from this research, several strategic recommendations are proposed. These recommendations aim to enhance the operational efficiency, safety, and resilience of first responder organizations that responds to disasters. The lessons learned from such a massive disaster that was named as *‘the disaster of the century’* is particularly important as Türkiye’s most populous city, Istanbul, are of particular significance given the city’s vulnerability to earthquakes. These recommendations offer valuable insights in the event of a possible scenario [50].

### Integrated communication and coordination systems

First responder organizations would benefit significantly from an integrated system that facilitates seamless communication and coordination. This system should include advanced messaging software, enabling real-time communication across different operational teams (interoperability), support for conference calls, and capabilities for sharing location data and other critical information. The European Commission’s support for nearly 14 different projects first responder technologies underlines the importance of such innovations in building disaster-resilient societies.

### Technology-enhanced training and protocols

Adopting common protocols and conducting joint training sessions among first responder organizations can harmonize operations and enhance teamwork. Projects like ASSISTANCE, which focus on situational awareness tools and tailored training scenarios, underscore the value of leveraging virtual reality (VR), augmented reality (AR), and mixed-reality technologies in preparing responders for the complexities of disaster management [44]. The FRs need to get culturally appropriate theoretical and practical training before entering real environments [51].

### Advanced technological support

The deployment of drone technology, wearable devices, and AI-enhanced software tools can revolutionize disaster response efforts. Drones, for instance, offer rapid and detailed survey capabilities of disaster zones, while wearable technology can monitor the health conditions of responders, detect environmental threats, and aid in decision-making processes. The TeamAware project, funded by the European Commission, exemplifies the potential of integrating such technologies to improve situational awareness and response capabilities [45]. Another good practice is the IProcure Security PCP project’s aims to enhance the triage process aligns with the emphasis on employing advanced technologies to streamline emergency response, resource management, and coordination [46].

### Real-time data analytics

Access to real-time data analytics tools is critical for enabling first responders to make informed and timely decisions. These tools can evaluate risks, optimize rescue operations, and prioritize resource allocation effectively. Incorporating data analytics into the operational framework can significantly enhance the effectiveness of disaster response efforts. It is suggested that researchers conduct simulation studies to design safe and secure emergency evacuation of crowded places as well as proper planning for better and faster access of aid squads to this location [52].

### Mental health support

Recognizing the emotional and psychological toll on first responders, there is a pressing need to integrate mental health support into the disaster management framework [53]. It is recommended to include ‘mental health’ to be one of the capability gaps identified by the International Forum for Advanced First Responder Innovation (IFAFRI), emphasizing the holistic well-being of responders [11].

### Cross-organizational collaboration

Disasters are chaotic situations [54]. Therefore, enhancing collaboration between first responders, local authorities, national agencies, non-governmental organizations, and international support teams is essential for a unified and effective disaster response. Multi-organizational collaboration enriches the pool of resources and expertise, facilitating a comprehensive approach to disaster management [55–57].

## Conclusion

This study fills an important gap in the literature by investigating the innovation needs and challenges faced by first responders during the 2023 earthquakes. These needs emphasise the importance of Integrated Communication and Coordination Systems, Technology Enhanced Training and Protocols, Advanced Technological Support, Real-Time Data Analytics, Mental Health Support, Inter-Institutional Collaboration and the need for innovations in this field. The importance of co-operation for the effective use of scarce resources and saving more lives has emerged. Innovations such as remote health and location monitoring, alongside tools that alert responders to potential risks, are crucial for minimizing the impact of disasters. Ultimately, embracing technological advancements is essential for enhancing disaster management and resilience, underscoring the importance of continued investment and accessibility in this field.

This study addresses a critical gap in disaster response research by systematically analysing the innovation needs and operational challenges faced by first responders during the 2023 earthquakes in Türkiye. Whilst prior studies have examined generic disaster response frameworks, the present research uniquely addresses the first-hand experiences of responders during the earliest and most chaotic phase of the disaster. By identifying key challenges such as fragmented communication systems, inadequate protective technologies and inadequate mental health support, this study provides a roadmap for improving disaster response mechanisms through targeted innovations and policy interventions. The findings emphasise the pressing need for integrated communication systems to facilitate seamless coordination between agencies, technology-enabled training to prepare responders for dynamic disaster environments, and advanced data-driven tools for real-time decision-making. Furthermore, the study emphasises the necessity of mental health support programmes, acknowledges the psychological burden on first responders and advocates for multi-organisational collaboration to optimise resource allocation and operational efficiency.

Future research should focus on the application and evaluation of these proposed innovations in real-world disaster scenarios. Simulation-based studies and pilot programmes integrating AI-assisted analyses, augmented reality training and next-generation protective equipment can provide empirical evidence on their effectiveness. In addition, interdisciplinary collaborations between disaster management experts, technology developers and policy makers are important to ensure that emerging innovations are not only technologically feasible but also practically applicable in high-risk emergency settings. As a result, this study reinforces the need for continued investment in disaster response innovation to increase resilience, minimise loss of life and protect first responders. The future of disaster management depends on using state-of-the-art technology, fostering collaboration and prioritising both physical and psychological safety; ensuring that first responders are equipped, protected and prepared for the increasing frequency and severity of disasters.

## Electronic supplementary material

Below is the link to the electronic supplementary material.

Supplementary Material 1

Supplementary Material 2

## Data Availability

Qualitative data are partly published as supplementary material. Fully anonymized datasets can be provided by the corresponding author upon reasonable request.

## Declarations

### Limitations

The study’s findings were derived from first responder teams responding on the first day of the 2023 Türkiye earthquake; however, the qualitative nature of the study restricts limits the transferability of these findings. While the study is based on Turkish experts, its results may be applicable to other similar cultural contexts. It is important to note that generalization in qualitative research, as emphasized by Smith [58] relies on analytical and inferential reasoning rather than statistical-probabilistic generalizability. In consideration of the correlation between the codes and subthemes identified in this study, which pertain to disaster management, it is evident that they exhibit interconnection in specific domains. However, the researchers’ reaching a consensus during the collaborative meetings served to mitigate this.
