# Supplementary material for: First responders’ experiences with major earthquakes in Türkiye: a qualitative study of innovation needs and challenges
Source: BMC Emerg Med. 2025 Apr 14;25:60. doi: 10.1186/s12873-025-01217-9 (PMC11998227; doi:10.1186/s12873-025-01217-9)
Supplement: Supplementary file 1 — Supplementary Material 1 [file 12873_2025_1217_MOESM1_ESM.docx]

**Supplementary Material**

Theme 1: Resources theme interview notes

| **Participant Code** | **Interview Notes** | |
| --- | --- | --- |
| **P2** | *“I have worked as a drone pilot in Disaster Coordination Centre. I have used 3 different drones: DJI Mini Pro3, MAVIC –with thermal camera- and 2-piloted grand drones…”* | |
| **P2** | *"... Sometimes the military would activate jammers for security reasons. This prevented us from communicating with the drones. We should be able to continue with other methods in such cases".* | |
| **P3** | *“Sound recording, camera, drone and thermal camera technologies were used. We used radios but communication between teams was weak. Everybody was focused on their own work.”* | |
| **P4** | *“We used helmets, fire suits, goggles, masks and gloves. The suit must be in two pieces for toilet use. Helmets must be undamaged. It is not safe to reuse damaged ones. Boot soles can melt. Goggles are useless because of the dust.”* | |
| **P5** | *"We had search and rescue equipment, sound recorders and cameras... We got help from Google and Yandex to get to the site. I worked in firefighters' uniforms because I thought they were more suitable. We also had office uniforms and rescue uniforms, which we also used. Our team continued with the same outfits; we did not use additional ones".* | |
| **P6** | *“… We had sound recorders, cameras, other rescue equipment and borescope camera for stairwells. First, we tried to rescue the living people. Then we tried to listen sound sources. However, it was problematic due to noise.”* | |
| **P7** | *"We worked in normal hospital clothes. We were cold because of the weather.* |  |
| **P9** | *"…. The car of the National Medical Rescue Team (UMKE) arrived and 5 of us went with it. We provided medical care with the equipment we had in our backpacks from the hospital.”* | |
| **P9** | *"We had no radio, so we had communication problems...”.* | |
| **P10** | *"We only had mobile phones. The messages came through WhatsApp. However, due to network problems, the location information either did not come or came late".”* | |
| **P11** | *"The UMKE outfits had been updated to turquoise, but as not all the outfits had been purchased, there was colour confusion in the field. Unity could not be maintained with red and turquoise. Endurance was more important than colour. Most of our colleagues' boots and clothes were torn. "* | |
| **P14** | *“We used regular firefighter outfits, helmet, glasses, mask and gloves.”* | |

Theme 2: Needs theme interview notes

| **Participant Code** | **Interview Notes** |
| --- | --- |
| **P2** | *“Thermal camera mounted drone was used like a hand camera as well. This device went into 20 meters underground, in debris…”* |
| **P2** | *"The building was in danger of collapsing. It was not safe, and no one could analyse it. So, we could not enter it. If we could determine the right place to drill a hole and get inside, we could rescue the people. The building was unrecognisable even to the people in the neighbourhood.”* |
| **P8** | *"The emergency room was very small, cold, crowded and there was no security. There were aftershocks, but we had to work rather than run away in those circumstances. If they could fly a few drones over Hatay or use satellites to survey the area, we could have known the magnitude... There were many sick and injured people, the emergency service was small, there was not enough equipment and there were not enough medical staff.”* |
| **P8** | *“In such huge disasters, medical care cannot be accomplished without 100 bed capacity field hospitals with EMT3 level training.”* |
| **P9** | *"We immediately started treating the injured, but there was no record of when and how the patients arrived.”* |
| **P10** | *"While working in the rubble, there was no safe field or security. A quick scan with drones was possible. Thermal cameras could have helped find living people. Drones could also have kept us safe. Where were we, were there any threats in the area?"* |
| **P14** | *"We did not work with the professional rescue teams. We worked with the volunteer rescuers. Lack of tools and medical equipment, lack of electronic registration system, inability to work with professional rescuers, lack of coordination and communication, challenges of transportation were harsh conditions, this was a huge disaster".* |

Theme 3: Collaboration theme interview notes

| **Participant Code** | **Interview Notes** |
| --- | --- |
| **P1** | *“I worked with Italian Teams and UMKE in Hatay…”.* |
| **P2** | *"On the first day of the earthquake, we travelled in an AKOM vehicle with three people. Then we went to Hatay. We started working with AFAD and exchanged mobile phone numbers. Then AFAD could send the notifications… We used WhatsApp to communicate with AFAD staff in the earthquake zone”.* |
| **P3** | *"... we knew the team we were working with, we knew our skills and techniques, but when a civilian rescue team arrived, sometimes we could not cope with their techniques...".* |
| **P4** | *"We arrived at Kahramanmaraş AFAD. Then we were taken to apartment X in district X. After taking over from AFAD, we were in the zone at 03.45. We started working within 24 hours of the first earthquake..."* |
| ***P5:*** | *"We were divided into two teams. Apart from the building we were assigned to, we took responsibility for another building where there was another group of volunteers. With our teammates, we tried to reach a resident in that building. They were the biggest help we could find."* |
| **P6** | *"... went there with 5 vehicles as 22 people... when we arrived, we went to AFAD... In the area where we were working, there was a UMKE team. We rescued people with the routine procedure. After UMKE entered, they took the necessary steps, such as putting on the drip or applying collar to the neck. We took the patient on the stretcher, let him out and handed him over to the ambulance team".* |
| **P7** | *“In the plane, there were AFAD, UMKE, Turkish Radio Amateur Society (TRAC) and military search and rescue teams. We landed on İncirlik Airbase at 16:30 next day.”* |
| **P8** | *“We were the first arriving group to the hospital as 1 emergency physican, 1 cardiovascular surgeon and 11 medical doctors with different branches.”* |
| **P9** | *"We decided to go to the earthquake zone ourselves on the 2^nd^ day, first we considered hitchhiking, then a UMKE vehicle arrived and 5 of us travelled with it".* |
| ***P9:*** | *"...lack of cooperation with professional rescue teams."* |
| **P10** | *"I am a UMKE staff member. The first two days I worked in the emergency service in the resuscitation room, triage and treatment and patient dispatch. The last two days I worked in a medical team of 5 people in the debris search and rescue zone”.* |
| **P10** | *"Since we were not given a specific task in the hospital, we split up to other units."* |
| **P11** | *"I am a UMKE staff member. In the Adıyaman centre we were included in the UMKE Medical Endpoint. There was AFAD, international and national volunteer teams who gave us an address and we went to the rubble zone. We worked in two teams of 5 people each”.* |
| **P13** | *"The health directors of Adıyaman and several provinces were completely changed before the earthquake.* |

Theme 4: Innovation theme interview notes

| **Participant Code** | **Interview Notes** |
| --- | --- |
| **P2** | *"Using a drone equipped with a thermal camera, it took 4-5 hours to rescue a human-shaped object emitting heat. Then it was understood that the object was not a baby, but a teddy bear. The situation caused a loss of time. It was understood that detecting the heat wave and shape was not enough. It looked like the silhouette of a baby, but it was a fibre heated by the sun. It was not enough to just detect the shape and heat. There is a need to add sensors that can detect parameters such as breathing rate, heartbeat or body movement".* |
| **P2** | *"... The damage assessment was done with drones within 15 minutes, whereas the area was discovered after 5-6 hours of pedestrian assessment. First, we did an aerial survey of the area. At the first stop, we could not see any living people, but we could see the dead. Drones were very useful to see the big picture".* |
| **P2** | *"The materials we use here have to be robust for harsh environments such as dust, collision, rain, iron or glass cutting. They have to be outdoor materials, not the ones we found on the market”.* |
| **P2** | *"Drone flying has to be done in all weather conditions, it has to be done indoors, and the drone lens has to be protected. It gives different results in sunny and cloudy weather... Drones need to have night vision and that is very important”.* |
| **P2** | *"In the areas where we were working, there could be a gas leak that could lead to an explosion. You might not be able to smell it because you can't smell anything because of the corpses and the intense carbon dioxide. Not only corpses, but also rotten food, exploded fridges and meat start to smell. Equipment that can diversify these odours is necessary.* |
| **P3** | *"Signal transmitters such as smart watches would make it easier to find people alive in the rubble. Helmets need to protect ears, eyes, nose and mouth. We have to solve the smell problem..."* |
| **P8** | *"You have to go to the hospital in the earthquake zone with your team. Cleaners, security, secretaries... Just sending doctors and nurses to the disaster area is not enough. Each hospital should have a 'core management team'. They should come from the unaffected towns and be deployed as a team. They can be defined by software, in advance. This way we know all the resources of the hospital we are working with before the disaster. The current hospital system cannot manage a disaster with only disaster management plans”.* |
| **P8** | *“The innovative triage systems must take photos, make identification, measure vital signs, and show patients’ location. I think medical first responders shall have nasal end-tidal CO_2_ measurement devices. It shows everything. Massimo has such a device. The bigger ones also perform hemogram analysis. Hundreds of patients can be saved with these devices. Identification is very important; hospitals should have put barcoded bracelets to the saved victims.”* |
| **P9** | *"In the hospital where we were working, there was some damage, but there was no analysis of the hospital construction. The aftershocks continued. As we did not have a tent, we had to continue our work in the emergency service in the effected hospital. If there had been equipment to analyse the safety of the buildings we were working in, we would have felt safer. We took too many risks. We could have been under the rubble due to aftershocks while we are working. …. We could have felt safer if we had wearable technology, sensors and drones”.* |
| **P10** | *“Clothes that we wore were not protective for threats under debris. There could be more protective helmets, boots, gloves and glasses. ... Clothes need to be changed more frequently. In each wash, they lose water-proof quality.”* |
| **P11** | *"When we entered the ruins, the safety of the building could not be guaranteed. We could not feel safe during the aftershocks. If the drones had been used effectively, the damage to the area could have been assessed and a faster rescue operation could have been carried out by using thermal cameras to locate people under the rubble".* |
| **P12** | *"The GPS trackers are necessary because when you send the team into the zone, there is always a risk of them being trapped under debris; we encountered this twice, once when the earthquake magnitude was 5.2 and the other when it was 4.8, we were 20 metres under the ground. If a building had collapsed on us, we could have been trapped inside”.* |
| **P12** | *"...challenges to find those who are still alive. We tried to listen with a device, but it was problematic due to the high level of noise. The main weakness of the device is that it requires silence. Even when we were silent, it detected the work next to ours. We need devices that can tell the difference between dead and alive. The dogs could not distinguish between smells because there were so many people.* |
| **P15** | *"When we asked if the hospital was safe, they told us that technical services and engineers had checked it and found some damage, but it was not critical. They said the service could continue. There were patients everywhere, they were lying on the floor, and they could not be drained. Amputees had to be transferred. There was no information and not enough ambulances. The open area of the hospital could not be used for the emergency".* |

Theme 5: Disaster management theme interview notes

| **Participant Code** | **Interview Notes** |
| --- | --- |
| **P5** | *"We were divided into two teams. Apart from the building we were assigned to, we took responsibility for another building where there was another group of volunteers. With our teammates, we tried to reach a resident in that building. They were the biggest help we could find".* |
| **P7** | *“I think there should have been a coordination headquarter from centre to the farthest edge, especially in Adana. Even if it was online or mobile…”.* |
| **P8** | *“The 'core management team' needs to know all the resources, roads, airports of that province and this needs to be prepared in advance. These will support the deployed teams to manage disasters”.* |
| **P9** | *“Cold, rain, crowd, lack of coordination, large number of injured, lack of tools and medical equipment, lack of electronic registration system, lack of cooperation with professional rescue teams...".* |
| **P10** | *"Since we were not given a specific task in the hospital, we split up to other units. The injured were treated immediately, but it was not known what their previous medical condition was, how old they were or where they came from. So, they could not be registered”.* |
| **P12** | *"We should have seen the 3D models of the original buildings at that moment. The sounds were mixed up, we could not tell which building was which and where we were in the original building... Search and rescue teams need to have access to a database of this information. We did not know how many people were in the building, how old they were, what their names were, or any information about the building. It was such a mess”.* |
| **P13** | *"The health directors of Adıyaman and several provinces were completely changed before the earthquake. This is not good for management. However, to compensate for this, a technological approach and cooperation can be developed. The managers were also victims; a software could be used to appoint other cities’ managers to help them".* |

Theme 6: Challenges theme interview notes

| **Participant Code** | **Interview Notes** |
| --- | --- |
| **P1** | *"And there was a relative of a tribal member who said, 'If you bring any construction equipment, we will kill you. This risk also arises because they think their relative is alive under the rubble".* |
| **P2** | *“If we were under the debris and another building collapsed on that, we could have been trapped…”.* |
| **P2** | *"In the areas where we were working, there could be a gas leak that could lead to an explosion. You might not be able to smell it because you can't smell anything because of the corpses and the intense carbon dioxide. Not only corpses, but also rotten food, exploded fridges and meat start to smell... Cold, rain, construction machinery, heat-emitting pieces of glass made things more difficult".* |
| **P3** | *"We did not know if the buildings we entered were safe. We also knew nothing about the safety of the environment, the amount of rubble, or the risks and dangers around us. We knew nothing, but we kept working”.* |
| **P3** | *"We were trying to drill a gallery and get into the rubble and a man came and said: 'We have an injured relative over there, you are working here but there are people out there. People came and said, 'I have a child over there', the other one came and said, 'My spouse is over there', if we look at both situations, please decide which one we should go to...".* |
| **P4** | *"One of the ruins we worked on was a half-collapsed building. I mean, 3 floors of a 6-storey building, shop, first and second floors collapsed, and the 4th, 5th and 6th floors were barely standing, with exploded columns, we were looking for a Syrian medical student there”.* |
| **P5** | *"We saw nothing until dawn. We were working in the dark and did not know how many of the buildings had been damaged. One of the risks was iron bars in the galleries we opened. These hurt the people we were trying to rescue; we tried to bend or cut them and then try to cover them”.* |
| **P8** | *"The people who came to the ambulance were not identifiable. There was no security. Someone came to me and said, 'This group came as volunteers,' but I did not let them in. I could not see who they really were”.* |
| **P9** | *"We took too many risks. We could have been under the rubble due to aftershocks while we are working. There was no safe space or security personnel to protect us when we were working in the rubble zone. We could have felt safer if we had wearable technology, sensors and drones”.* |
| **P10** | *"Cold, rain, crowds, uncoordinated distribution of tasks, many injured people... It would give a sense of safety and security if our location information and vital signs were monitored".* |
| **P11** | *“There were too many challenges, such as lack of communication and coordination, working in the dark…”* |
| **P12** | *"Danger of buildings collapsing, dust, aftershocks, injuries, challenges in finding the survivors... We tried to listen with equipment, but it was problematic because of the high noise levels."* |
| **P14** | *"Damage caused by iron bars, injuries caused by tiles, cuts caused by glass were too frequent... Long working hours, infections of the eyes caused by dust and particles, impaired eyesight, blocked ears, especially enervation caused by children's bodies, stress..."* |
