# Supplementary material for: First responders’ experiences with major earthquakes in Türkiye: a qualitative study of innovation needs and challenges
Source: BMC Emerg Med. 2025 Apr 14;25:60. doi: 10.1186/s12873-025-01217-9 (PMC11998227; doi:10.1186/s12873-025-01217-9)
Supplement: Supplementary file 2 — Supplementary Material 2 [file 12873_2025_1217_MOESM2_ESM.docx]

**Supplementary Material**

Turkish to English Translate Form

Interview Form

*“Faced by First Responder Teams in Earthquake Response and Their Innovation Needs"*

1. Can you tell us a bit about yourself?
   *(Guiding Questions: Can you provide information about the institution you work for, your role, and your years of experience?)*
2. How did you reach the disaster site after the earthquake?
   *(Guiding Questions: What challenges did you face during transportation? In which areas do you think improvements or innovations are needed in this process?)*
3. How was communication at the disaster site?
   *(Guiding Questions: Can you describe the difficulties you encountered while communicating with teams and different institutions? What innovations do you think are needed to improve communication?)*
4. What do you think about the technologies you used?
   *(Guiding Questions: What technologies were used at the disaster site? Were they sufficient? Were there any technological shortcomings or areas that needed improvement?)*
5. How was your situational awareness?
   *(Guiding Questions: What information were you able to use to understand and assess the disaster situation? What information gaps or uncertainties did you face? What innovations are needed to address these issues?)*
6. How would you evaluate the teamwork process?
   *(Guiding Questions: What were your experiences working in collaboration with different units? What challenges did you face in terms of coordination? What innovations could be beneficial to enhance collaboration?)*
7. What are your thoughts on protective equipment and clothing?
   *(Guiding Questions: Were the existing equipment and clothing sufficient? In what situations did you experience deficiencies? What kinds of innovations are needed in protective materials?)*
8. What were the biggest challenges you faced during the disaster response process?
   *(Guiding Questions: What shortcomings did you feel during this process? What technological or structural changes do you think should be made for better responses in the future?)*
9. In your opinion, what are the most important areas for improvement in disaster management processes?
   *(Guiding Questions: Can you share the areas you see as most critical? What systems or tools do you think should be developed for the future?)*
10. What were the most important lessons you learned from this experience?
    *(Guiding Questions: What aspects do you think should be focused on to be better prepared in the future?)*

Orijinal Form:

“İlk Müdahaleci Ekiplerin Depremlere Müdahalesinde Yaşadıkları Zorluklar ve İnovasyon Gereksinimlerinin Değerlendirilmesi”

1. Bize biraz kendinizden bahseder misiniz?

(Yardımcı Soru Cümleleri: Çalıştığınız kurum, göreviniz ve deneyim süreniz hakkında bilgi verebilir misiniz?)

1. Deprem sonrası olay yerine nasıl ulaştınız?

(Yardımcı Soru Cümleleri: Ulaşım sürecinde karşılaştığınız zorluklar nelerdi? Bu süreçte hangi konularda iyileştirme ya da yenilik ihtiyacı olduğunu düşünüyorsunuz?)

1. Olay yerinde iletişim nasıldı?

(Yardımcı Soru Cümleleri: Ekipler ve farklı kurumlar arasında iletişim kurarken karşılaştığınız zorlukları anlatabilir misiniz? Sizce iletişimin geliştirilmesi için hangi inovasyonlara ihtiyaç var?)

1. Kullandığınız teknolojiler hakkında ne düşünüyorsunuz?

(Yardımcı Soru Cümleleri: Olay yerinde hangi teknolojiler kullanıldı? Bunlar yeterli miydi? Teknolojik açıdan eksiklikler veya geliştirilmesi gereken alanlar var mıydı?)

1. Durumsal farkındalığınız nasıldı?

(Yardımcı Soru Cümleleri: Afet alanındaki durumu anlamak ve değerlendirmek için hangi bilgileri kullanabildiniz? Karşılaştığınız bilgi eksiklikleri veya belirsizlikler nelerdi? Bunları gidermek için ne gibi yeniliklere ihtiyaç duyuluyor?)

1. Ekip çalışması sürecini nasıl değerlendirirsiniz?

(Yardımcı Soru Cümleleri: Farklı birimlerle iş birliği yaparken yaşadığınız deneyimler nelerdi? Koordinasyon açısından hangi zorluklarla karşılaştınız? İş birliğini artırmak için hangi inovasyonlar faydalı olabilir?)

1. Koruyucu ekipmanlar ve kıyafetler konusunda ne düşünüyorsunuz?

(Yardımcı Soru Cümleleri: Mevcut ekipman ve kıyafetler yeterli miydi? Hangi durumlarda eksiklik yaşadınız? Koruyucu malzemeler açısından ne tür yeniliklere ihtiyaç var?)

1. Genel olarak afet müdahale sürecinde karşılaştığınız en büyük zorluklar nelerdi?

(Yardımcı Soru Cümleleri: Bu süreçte hangi eksiklikleri hissettiniz? Gelecekte daha iyi bir müdahale için hangi teknolojik veya yapısal değişikliklerin olması gerektiğini düşünüyorsunuz?)

1. Sizce afet yönetimi süreçlerinde en önemli iyileştirme alanları neler?

(Yardımcı Soru Cümleleri: En kritik gördüğünüz alanları paylaşabilir misiniz? İleriye dönük olarak hangi sistem veya araçların geliştirilmesi gerektiğini düşünüyorsunuz?)

1. Bu deneyiminizden öğrendiğiniz en önemli dersler neler oldu?

(Yardımcı Soru Cümleleri: Gelecekte daha iyi hazırlanmak için hangi konulara odaklanılması gerektiğini düşünüyorsunuz?)
